# Mobility Requirements and Joint Loading during Straight Walking and 90° Turns in Healthy Older People and Those with Hip Osteoarthritis

**DOI:** 10.3390/jcm13175021

**Published:** 2024-08-25

**Authors:** Hannah Steingrebe, Stefan Sell, Thorsten Stein

**Affiliations:** 1 Institute of Sports and Sports Science, Karlsruhe Institute of Technology, 76131 Karlsruhe, Germany; stefan.sell@kit.edu (S.S.); thorsten.stein@kit.edu (T.S.); 2 Joint Center Black Forest, Hospital Neuenbuerg, 75305 Neuenbuerg, Germany

**Keywords:** turning, hip mobility, gait analysis, range of motion, joint loading

## Abstract

**Background/Objectives**: Hip mobility and joint loading in hip osteoarthritis (HOA) patients are mostly assessed during straight walking. Yet, mobility limitations in the frontal and transverse planes are rarely found during this task in subjects with mild-to-moderate symptoms. Turning movements are frequently encountered during everyday life and might require larger hip mobility compared to straight walking, especially in the frontal and transverse planes. Thus, hip mobility and hip loading during straight walking and 90° turns in persons with HOA and healthy older adults were compared in this study. **Methods**: A retrospective analysis was conducted on 21 subjects with mild-to-moderate HOA and 21 healthy controls. Hip angles and moments were assessed during straight walking and 90° step and spin turns. Gait analysis was conducted using a motion capture system and a force plate. Group and movement task differences were assessed with a mixed-model ANOVA. **Results**: Peak abduction and adduction angles were largest during the step and spin turn, respectively, as were the group differences between HOA subjects and healthy subjects. Both turns require a greater transverse hip range of motion compared to straight walking. Limitations in transverse hip mobility in the HOA group were especially prominent during the step turn. Both turns cause higher joint moments than straight walking. **Conclusions**: The additional inclusion of 90° step and spin turns into gait analysis can enhance early identification of hip mobility limitations in the frontal and transverse planes in subjects with mild-to-moderate hip osteoarthritis. Early diagnosis is crucial for the timely application of conservative treatment strategies.

## 1. Introduction

Patients with hip osteoarthritis (HOA) suffer from pain and a loss of function leading to a decrease in quality of life [1]. Functional loss is mainly caused by muscle weakness [2] and limitations in passive and active hip range of motion (ROM) [3]. Previous analyses of active hip ROM have mainly focused on straight walking [4,5,6]. Likewise, joint moments, as a surrogate measure of joint loading, are also mostly assessed during straight walking. In a meta-analysis on HOA-induced changes in peak hip flexion and adduction moments during walking, differences were found for subjects with severe HOA but not for those with only mild-to-moderate symptoms [7]. However, joint mobility requirements and joint loading in terms of external moments might be larger during other movements of daily living, especially in the frontal and transverse planes.

One task that has gained attention from various research fields is turning or curve walking [8,9,10,11]. Turning movements are encountered frequently during activities of daily living as 35–45% of daily steps are turning or nonlinear steps [12], and turns are a risk factor for falls [13]. Turning movements can be conducted either in a spin or step turn manner [14]. Both movement patterns require more pronounced external rotation of the hip; step turning requires larger hip abduction, and spin turning requires larger hip adduction than straight walking [15]. Yet, these biomechanical insights have been obtained in a sample of healthy young adults. In HOA patients, limited passive internal rotation and adduction are often core criteria for a functional HOA diagnosis [16], and limitations in external rotation mobility have been associated with higher levels of disability [17]. To the best of our knowledge, only one study of people with HOA included turning movements; this showed decreased peak hip abduction and adduction as well as peak hip adduction moments during 45° turns compared to age-matched healthy controls [18]. However, knowledge of transverse plane biomechanics is still lacking, and ROM requirements and joint moments of different movement tasks have not been compared. Additionally, it has been found that the majority of turns during daily life are between 76° and 120° [19], meaning that turns in this range presumably are of higher practical relevance.

Therefore, this study aimed to compare hip mobility, in terms of ROM and peak angles, and joint loading, in terms of peak external hip moments, between straight walking and 90° step and spin turns in healthy older adults and in subjects with mild-to-moderate HOA. We hypothesised that 90° turns would require larger peak hip angles and overall hip ROM and would provoke higher joint moments in the frontal and transverse planes compared to straight walking. Subsequently, we hypothesised that mobility deficits as well as modifications in the joint loading of HOA subjects would be more pronounced during turning than during straight walking.

## 2. Materials and Methods

In this secondary analysis, data from a study on the effects of hip bracing in different movement tasks were reanalysed. Therefore, the data have been published in part elsewhere [19].

### 2.1. Participants

Subjects for this study were recruited from local physiotherapy practices, via local newspapers, or from the university community and thus represent a sample of convenience. In total, 42 subjects participated in this study, including 21 subjects with mild-to-moderate unilateral primary HOA (10 females, age: 64.0 ± 9.6 years; body mass index (BMI): 24.2 ± 2.9; Harris Hip Score (HHS): 74.6 ± 11.8) and 21 healthy participants (10 females, age: 63.1 ± 9.2 years; BMI: 25.2 ± 2.7; HHS: 98.4 ± 2.3). Subject groups did not differ regarding age, height, body mass, or BMI. Additionally, subjects from the control group were randomly and counterbalanced assigned to a left or right leg group to match the total number of affected right and left limbs of the HOA group. Subjects in the HOA group had radiologically confirmed HOA of Kellgren–Lawrence grade 2 or higher, hip pain during activities of daily living and decreased hip function (HHS ≤ 95). The healthy subjects had no radiological signs of HOA (Kellgren–Lawrence Score ≤ 1), did not experience hip pain during activities of daily living, and had good hip function (Harris Hip Score > 95). Detailed inclusion and exclusion criteria can be found in Table 1. The study procedure was approved by the ethics committee of the Karlsruhe Institute of Technology. All participants gave their written informed consent before study participation.

### 2.2. Testing Protocol

**Figure 1:**
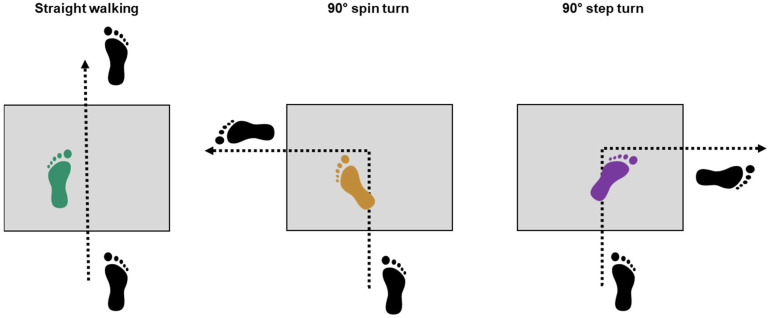
Schematic representation of foot placement during straight walking, 90° spin turn, and 90° step turn. Coloured feet represent affected limbs in hip osteoarthritis (HOA) subjects and matched limbs in healthy subjects. Green = straight walking, purple = 90° step turn, and yellow = 90° spin turn.

Subjects performed straight walking and pre-planned 90° step and spin turns (Figure 1) at a self-selected speed. We chose 90° turns due to their high practical relevance as right-angle turns are frequently encountered in urban settings [20], and the widespread usage of this turn angle in previous studies [15,21,22], and their inclusion in functional tests [23]. Movement velocity was controlled using light barriers and kept constant (within ± 5% of the first trial) across trials of the same movement task. For each movement task, five valid trials were recorded. During the turning trials, subjects initiated the turn within a marked corridor of 50 cm on the force plate. To standardize movement execution, subjects were instructed to perform the turn in an abrupt manner, resulting in an entire 90° turn within one gait cycle [24]. Turning was always conducted with the affected limb for the HOA subjects and a matched limb for the control group (Figure 1).

### 2.3. Gait Analysis and Data Processing

Whole-body movement was assessed with a 16-camera motion capturing system (200 Hz; Vicon Motion Systems, Oxford Metrics Group, Oxford, UK). Simultaneously, ground reaction forces (GRFs) were recorded using a 3D force plate (1000 Hz; Advanced Mechanical Technology Inc., Watertown, MA, USA). Kinematic and GRF data were filtered using a 4th-order Butterworth low-pass filter with a cut-off frequency of 15 Hz. A full-body marker set with 42 retroreflective markers was applied [25]. Three-dimensional hip joint angles and external joint moments were calculated using an inverse kinematics and dynamics approach with the multi-body ALASKA Dynamicus model [20] including the hip joint centre definition proposed by Harrington et al. [26]. Peak hip joint angles and moments as well as hip ROM during the stance phase of straight walking or the turning step of 90° turns were assessed. Additionally, forward-oriented centre of mass (COM) velocity at initial contact (IC) and toe off (TO) as well as stance phase duration were analysed. Mean values across the five trials of each movement were calculated for each subject. For five subjects, one trial of step or spin turning was detected as invalid after the measurement. These trials were excluded from the analysis, and the mean value was calculated across the remaining 4 trials.

### 2.4. Statistical Analysis

As the basis for this manuscript is secondary data, the a priori calculation of the sample size was based on effects induced by hip bracing during level walking in HOA subjects [27]. Calculations were conducted using G*Power (version 3.1.9.3) [28]. With observed effect sizes of 0.92 and 1.17 for peak hip adduction and internal rotation [27], a significance criterion of α = 0.05, and power of 0.95, the minimum sample size required was 18 and 12, respectively.

All statistical analyses were conducted using R (version 4.2.2) [29]. Normal distribution and homogeneity of variance were tested using the Shapiro–Wilk and Levene tests, respectively. Statistical differences were assessed using a mixed-model ANOVA with movement task (straight walking, step turn, spin turn) as the within-subject factor and group (healthy, HOA) as the between-subject factor. A Mauchly test was used to assess sphericity. If sphericity was violated, Greenhouse–Geisser estimates were used. Post hoc analyses were conducted using *t*-tests for dependent samples with Bonferroni corrections. The level of significance was set a priori to ɑ < 0.05. Partial eta squared (η_p_^2^) was used for effect size estimation.

## 3. Results

**Figure 2:**
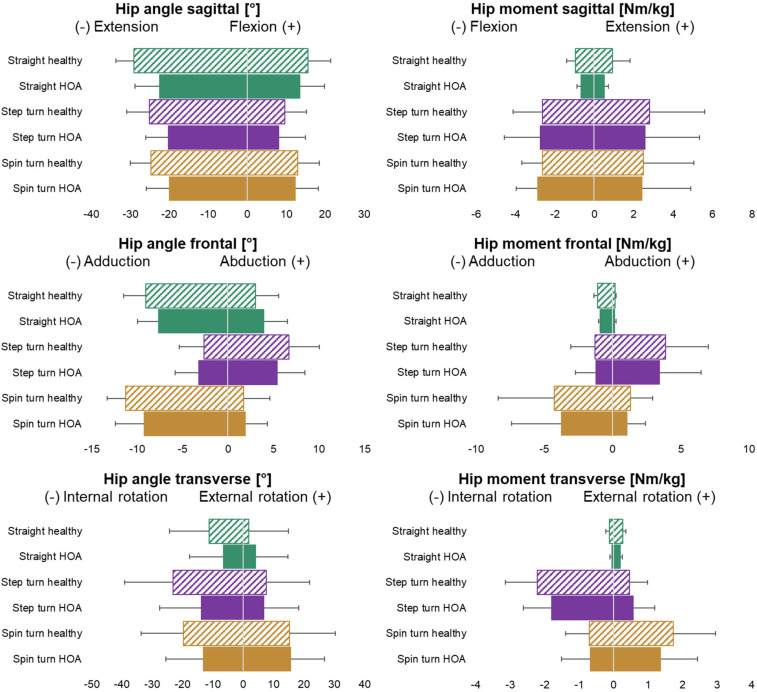
Mean and SD of peak hip angles [°] and external hip joint moments [Nm/kg]. Green = straight walking, purple = 90° step turn, and yellow = 90° spin turn. Solid bars = HOA subjects; hatched bars = healthy subjects.

**Figure 3:**
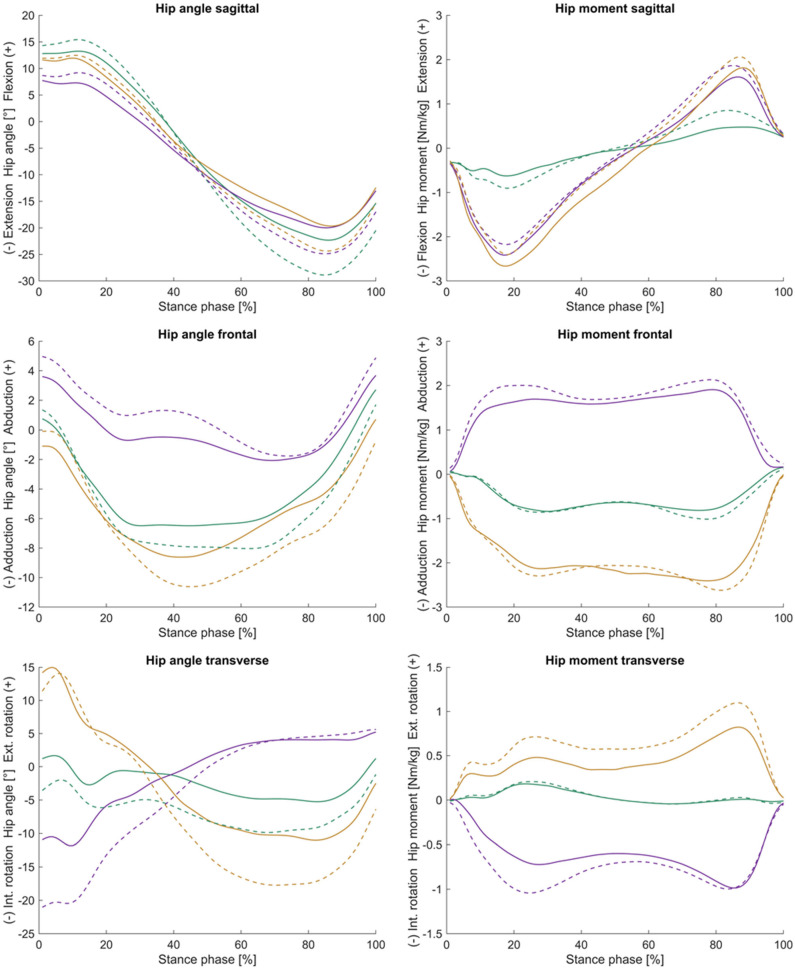
Sagittal, frontal, and transverse hip joint angle [°] and external hip joint moment [Nm/kg] time curves (mean) across the stance phase [%]. Green = straight walking, purple = 90° step turn, and yellow = 90° spin turn. Dashed line = healthy subjects; solid line = HOA subjects.

Means and standard deviations of spatio-temporal parameters are presented in Table 2, kinematic parameters are presented in Table 3, and kinetic parameters are presented in Table 4. Additionally, hip joint mobility and joint dynamics are visualised in Figure 2. Hip joint angle and moment time curves in the sagittal, frontal, and transverse planes are presented in Figure 3. The results from all statistical analyses can be found in Tables S1 and S2 in the Supplementary Materials.

### 3.1. Mobility Requirements of the Movement Tasks

For all kinematic parameters, namely peak hip angles and hip ROM, a significant main effect for movement task (*p* < 0.001; η_p_^2^ 0.39–0.91) was found. Post hoc comparisons showed that straight walking and the step turn differed significantly (*p* < 0.001) for all kinematic parameters, with lower sagittal hip mobility and hip adduction but larger hip abduction and transverse mobility during the step turn. Likewise, straight walking and the spin turn differed significantly in all kinematic parameters (*p* < 0.001) except for frontal hip ROM (*p* = 1.00), with lower sagittal hip mobility and hip abduction but larger hip adduction and transverse mobility. Step and spin turns differed significantly (*p* < 0.001) for all kinematic parameters except for peak hip extension (*p* = 1.00) and peak hip internal rotation angle (*p* = 0.16). Thereby, lower hip flexion, sagittal hip ROM, hip adduction, frontal hip ROM, external rotation, and transverse hip ROM but larger hip abduction were found for the step turn compared to the spin turn.

### 3.2. Joint Loading during the Movement Tasks

For all kinetic parameters, namely peak external hip joint moments, a significant main effect for movement task (*p* ≤ 0.006; η_p_^2^ 0.16–0.78) was found. Pairwise comparisons showed that straight walking and the step turn differed significantly for all kinetic parameters (*p* ≤ 0.004) except for peak hip adduction moment (*p* = 0.91), with higher hip joint moments during the step turn. Likewise, straight walking and the spin turn differed significantly in all parameters (*p* < 0.001), with higher joint moments during the spin turn. Step and spin turns differed significantly (*p* < 0.001) in frontal and transverse peak joint moments, with the step turn provoking higher peak hip abduction and internal rotation moments but lower hip adduction and external rotation moments.

### 3.3. Effect of Hip Osteoarthritis on Hip Mobility and Joint Loading

**Figure 4:**
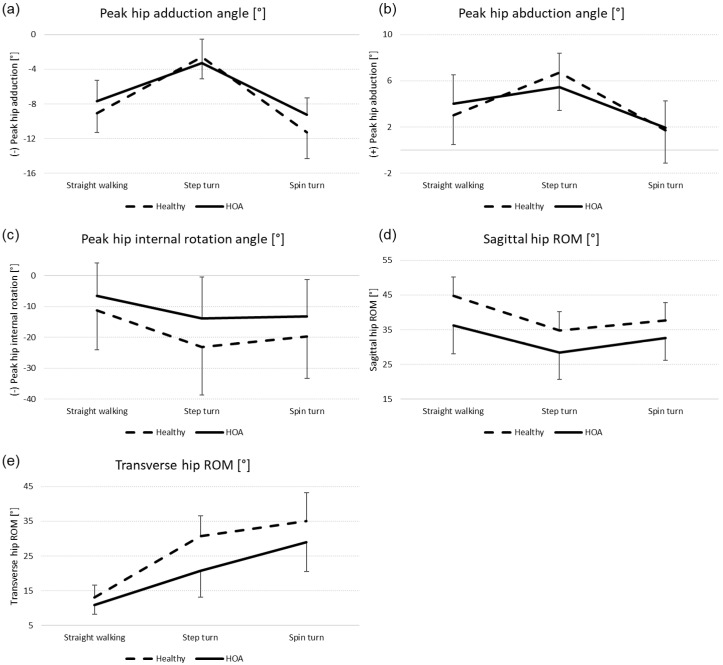
Mean and standard deviation of parameters with significant interaction effect of group and movement task: (**a**) peak hip adduction angle, (**b**) peak hip abduction angle, (**c**) peak hip internal rotation angle, (**d**) sagittal hip ROM, and (**e**) transverse hip ROM. Dashed line = healthy subjects; solid line = HOA subjects.

A significant main effect for group was found for peak hip extension angle (*p* = 0.002; η_p_^2^ = 0.22), sagittal hip ROM (*p* = 0.001; η_p_^2^ = 0.24), and transverse hip ROM (*p* < 0.001; η_p_^2^ = 0.28), with lower values in the HOA group. Significant interaction effects were found for peak hip adduction angle (*p* < 0.001), peak hip abduction angle (*p* = 0.001), peak hip internal rotation angle (*p* = 0.046), sagittal hip ROM (0.041), and transverse hip ROM (0.006) (Figure 4).

## 4. Discussion

To the best of our knowledge, this is the first study to compare 3D hip kinematics and dynamics during straight walking and 90° turns while walking in healthy subjects and those with mild-to-moderate HOA.

We expected turning to require larger frontal and transverse hip mobility and provoke larger hip joint moments in these movement planes. In line with this hypothesis, both 90° turns required larger hip external and internal rotation and overall transverse hip ROM. In contrast to our expectations, frontal hip ROM was lower (step turn) or no different (spin turn) than during straight walking. However, there was reduced adduction but increased abduction during the step turn and reduced abduction but increased adduction during the spin turn (Figure 2). As hypothesised, peak external hip joint moments in all movement planes were higher during both 90° turns than during straight walking, except for the peak hip adduction moment during the step turn. Thereby, observed joint moments are comparable to those reported for running [30], even though both turns are conducted with a lower COM velocity compared to straight walking.

Secondly, we expected mobility deficits and modifications of joint loading in HOA subjects to be more pronounced during turning than during straight walking. HOA subjects demonstrated decreased transverse ROM across all movements compared to healthy subjects. Thereby, group differences in transverse ROM and peak hip internal rotation are largest during the 90° step turn (Figure 4). While Taylor et al. [15] also reported larger external rotation angles during turning than during straight walking, they did not find increased hip internal rotation during turning. These differences in hip rotation might stem from differences in foot placement strategies used by younger and older subjects. This warrants further investigation.

In the frontal plane, HOA-induced limitations in hip adduction and abduction were most noticeable during the spin and step turn, respectively (Figure 4). Previous studies found no differences in peak hip ad- or abduction during straight walking between healthy subjects and those with mild or moderate HOA [5,31] and differences were only partly found for those with severe HOA [18,31,32,33]. Likewise, no overall effect of HOA on frontal plane kinematics was found in this study. Thereby, frontal hip ROM during straight walking and the gait velocity observed in the present study were comparable to those reported by Rutherford et al. [31], with about 9° and 1.33 m/s. However, significant interaction effects in our study showed that group differences in peak hip adduction and abduction were most noticeable during spin and step turns, respectively. Tateuchi et al. [18] also reported reduced abduction during a 45° step turn and reduced adduction during a 45° spin turn. However, subjects in their study suffered from end-stage HOA. Thus, analysing 90° step and spin turns might allow for the detection of frontal plane hip mobility limitations at an earlier disease stage and thus facilitate earlier treatment initiation.

Although joint moments were larger during the 90° turning movements, no overall group differences or interactions were found. Yet, large individual heterogeneity was observed for the joint moments during both 90° turns (Figure 2). These differences are likely to stem from different movement strategies adopted to perform the change in direction as rapidly as possible. Turning usually requires a leaning-in motion to counteract the centripetal force [8] which results in an increased distance between the base of support and the COM. Previous studies found that older adults reduce leaning-in motion in comparison to young adults [34] and that higher levels of knee extension strength are related to better maintenance of top-down segment reorientation and leaning-in behaviour in healthy older adults [21]. Thus, individual differences in hip pain or strength ability likely influence trunk lean and subsequently hip joint moments. Further analyses of whole-body movement strategies could identify coping strategies adopted by HOA subjects and identify those with more favourable hip joint loading to serve as a basis for gait retraining protocols.

For the sagittal plane, both turning movements required lower peak angles and less hip ROM than during straight walking (Figure 2). Nevertheless, reduced peak hip extension leading to decreased sagittal ROM was observed across all movement tasks for subjects with HOA. Yet, group differences for sagittal hip ROM were largest during straight walking (Figure 4). Thus, limitations in sagittal plane hip mobility are best evaluated during straight walking.

### Limitations

The results from this study have to be interpreted with regard to the following limitations. While transverse plane biomechanics represent important data for the analysis of turning movements, it has to be borne in mind that multi-body modelling is prone to errors in this plane. However, even if the modelling output might contain inaccuracies, the observed differences between movement tasks and groups are large.

Secondly, observed hip angles in the sagittal plane are shifted towards lower flexion and more pronounced extension compared to the literature. This offset is caused by differences in the definition of the pelvis neutral position [35,36]. Consequently, when comparing our data to other studies, individual model definitions should be considered.

Moreover, in our study, subjects were asked to perform the turns in an abrupt manner, as previously done in other studies [34], to increase standardization of the turning movement across trials and subjects. However, performing the entire 90° turn within one gait cycle might not reflect natural turning behaviour [37,38]. As joint moments [39], step width [38], and likely also joint angles increase with increasing turning angle, the peak angles and moments observed in our study might be larger than during turns in which the rotation of the body is split into multiple steps.

Further, to increase uniformity across multiple trials of the same subject, movement velocity was controlled using light barriers. While this approach allows for the detection of changes in movement velocity immediately during data acquisition, it does not guarantee consistent movement across all trials as only the mean velocity between the light barriers is evaluated. Therefore, averaging peak values across different trials might not represent peak values observed during individual trials. Thus, our results likely represent hip mobility and joint loadings across different movement strategies, and future analyses of whole-body movements on single-trial data are needed to clarify the role of different movement strategies on mobility requirements and joint loading.

Likewise, our analysis of the implications of mild-to-moderate HOA was performed at the group level to detect overall changes in turning biomechanics in this population. However, individual differences in HOA severity or sex might influence movement execution, as has been previously shown for straight walking [6,40].

Lastly, in our study, joint kinematics were only analysed during the stance phase of walking and turning to match the gait phase evaluated for joint dynamics. While this approach follows previous studies [15,18], analyses of joint kinematics during the entire gait cycle might add additional insights on turning movements.

## 5. Conclusions

In this study, we demonstrated that 90° turns require overall larger transverse hip mobility and task-specific increases in frontal hip mobility. Thereby, frontal and transverse plane mobility limitations of patients with mild-to-moderate HOA were more prominent during 90° turns than during straight walking. Thus, the routine inclusion of 90° turns during gait analysis might allow for the identification of frontal and transverse hip mobility deficits at an early disease stage of HOA and might therefore enable earlier access to conservative treatment options. Lastly, both turns cause larger joint moments at the hip joint than straight walking, with large inter-individual variability. The identification of individual movement strategies could be used as a foundation for gait retraining interventions.

## Figures and Tables

**Table 1:** Inclusion and exclusion criteria.

Group	Inclusion Criteria	Operationalisation
HOA	Radiologically confirmed HOA	Kellgren–Lawrence Score (K-L Score) of 2–4
	Symptomatic HOA	Hip pain during activities of daily living within the last 3 months
	Decreased hip function	Harris Hip Score between 65 and 95
	Unilateral HOA	Contralateral K-L Score ≤ 2; contralateral hip pain free within the last 3 months; unrestricted passive ROM of contralateral hip (sagittal ROM ≥ 90°; transverse ROM ≥ 15°; peak abduction ≥ 20; flexing contracture ≤ 10°)
Healthy	No radiological signs of HOA	Bilateral K-L Score ≤ 1 (if radiographic images available)
	No symptoms of HOA	No hip pain within the last 3 months during activities of daily living
	Good hip function	Harris Hip Score ≥ 96
**Group**	**Exclusion Criteria**	
HOA	Secondary HOA caused by trauma	
	Contraindications of X-ray imaging	
Both	BMI ≥ 35 kg/m^2^	
	Orthopaedic injury of other joints of the lower limbs and back (e.g., pain, osteoarthritis > grade 1 (self-reported), endoprosthesis, rheumatoid arthritis, acute herniated disc, etc.)	
	Neuromuscular disorders or neurological complaints (e.g., vertigo)	

**Table 2:** The mean (SD) of the evaluated spatio-temporal parameters during the stance phase of straight walking, a 90° step turn, and a 90° spin turn for healthy subjects and subjects with HOA. IC = initial contact; TO = toe off; COM = centre of mass.

Parameter	Straight Walking		90° Step Turn		90° Spin Turn	
	Healthy	HOA	Healthy	HOA	Healthy	HOA
COM velocity IC [m/s]	1.36 (0.12)	1.27 (0.19)	0.97 (0.12)	0.94 (0.14)	0.96 (0.12)	0.92 (0.19)
COM velocity TO [m/s]	1.39 (0.12)	1.29 (0.19)	0.98 (0.15)	0.94 (0.16)	0.97 (0.11)	0.93 (0.12)
Stance phase duration [s]	0.64 (0.05)	0.65 (0.05)	0.70 (0.08)	0.73 (0.10)	0.74 (0.08)	0.78 (0.14)

**Table 3:** The mean (SD) of the evaluated kinematic parameters during the stance phase of straight walking, a 90° step turn, and a 90° spin turn for healthy subjects and subjects with HOA. ROM = range of motion.

Parameter	Straight Walking		90° Step Turn		90° Spin Turn	
	Healthy	HOA	Healthy	HOA	Healthy	HOA
Peak hip flexion [°]	15.70 (5.75)	13.69 (6.14)	9.64 (5.58)	8.18 (6.84)	12.95 (5.55)	12.49 (5.82)
Peak hip extension [°]	−29.11 (4.5)	−22.56 (6.28)	−25.13 (5.76)	−20.32 (5.73)	−24.72 (5.25)	−20.08 (5.82)
Sagittal hip ROM [°]	44.81 (5.52	36.25 (8.34)	34.77 (5.58)	28.50 (8.01)	37.66 (5.25)	32.57 (6.49)
Peak hip abduction [°]	3.02 (2.58)	4.02 (2.56)	6.71 (3.37)	5.47 (2.98)	1.71 (2.92)	1.95 (2.38)
Peak hip adduction [°]	−9.04 (2.40)	−7.66 (2.30)	−2.61 (2.76)	−3.26 (2.53)	−11.25 (2.03)	−9.26 (3.12)
Frontal hip ROM [°]	12.06 (1.60)	11.68 (2.69)	9.32 (2.41)	8.73 (2.39)	12.97 (1.81)	11.21 (3.00)
Peak hip ext. rotation [°]	1.90 (13.2)	4.34 (10.5)	7.68 (14.3)	6.97 (11.5)	15.41 (15.0)	15.82 (11.1)
Peak hip int. rotation [°]	−11.19 (13.1)	−6.59 (11.00)	−23.07 (16.0)	−13.82 (13.7)	−19.62 (13.9)	−13.20 (12.2)
Transverse hip ROM [°]	13.08 (3.56)	10.93 (2.82)	30.75 (5.97)	20.79 (7.85)	35.03 (8.45)	29.02 (8.72)

**Table 4:** The mean (SD) of the evaluated kinetic parameters during the stance phase of straight walking, a 90° step turn, and a 90° spin turn for healthy subjects and subjects with HOA.

Parameter	Straight Walking		90° Step Turn		90° Spin Turn	
	Healthy	HOA	Healthy	HOA	Healthy	HOA
Peak hip extension moment [Nm/kg]	0.93 (0.90)	0.53 (0.19)	2.82 (2.80)	2.61 (2.74)	2.50 (2.58)	2.45 (2.46)
Peak hip flexion moment [Nm/kg]	−0.96 (0.43)	−0.69 (0.18)	−2.63 (1.48)	−2.77 (1.80)	−2.62 (1.06)	−2.89 (1.06)
Peak hip abduction moment [Nm/kg]	0.20 (0.08)	0.19 (0.09)	3.89 (3.13)	3.50 (3.01)	1.33 (1.62)	1.09 (1.35)
Peak hip adduction moment [Nm/kg]	−1.10 (0.25)	−0.92 (0.10)	−1.29 (1.75)	−1.25 (1.47)	−4.28 (4.10)	−3.78 (3.63)
Peak hip ext. rotation moment [Nm/kg]	0.26 (0.09)	0.20 (0.06)	0.46 (0.52)	0.58 (0.61)	1.74 (1.22)	1.37 (1.06)
Peak hip int. rotation moment [Nm/kg]	−0.12 (0.11)	−0.07 (0.04)	−2.21 (0.94)	−1.81 (0.81)	−0.71 (0.69)	−0.69 (0.83)

## Data Availability

The data presented in this study are only available on request from the corresponding author due to legal reasons.

## Supplementary Materials

The following supporting information can be downloaded at: https://www.mdpi.com/article/10.3390/jcm13175021/s1; Table S1: *p*-values of Shapiro-Wilk tests, Levene tests and mixed-model ANOVA; Table S2: Bonferroni corrected *p*-values from pairwise comparisons of significant ANOVA main effects for movement task using dependent sample *t*-tests.

## References

[1] Salaffi F., Carotti M., Stancati A., Grassi W. (2005). Health-related quality of life in older adults with symptomatic hip and knee osteoarthritis: A comparison with matched healthy controls. Aging Clin. Exp. Res..

[2] Loureiro A., Constantinou M., Diamond L.E., Beck B., Barrett R. (2018). Individuals with mild-to-moderate hip osteoarthritis have lower limb muscle strength and volume deficits. BMC Musculoskelet. Disord..

[3] Baker M., Moreside J., Wong I., Rutherford D.J. (2016). Passive hip movement measurements related to dynamic motion during gait in hip osteoarthritis. J. Orthop. Res..

[4] Leigh R.J., Osis S.T., Ferber R. (2016). Kinematic gait patterns and their relationship to pain in mild-to-moderate hip osteoarthritis. Clin. Biomech..

[5] Constantinou M., Loureiro A., Carty C., Mills P., Barrett R. (2017). Hip joint mechanics during walking in individuals with mild-to-moderate hip osteoarthritis. Gait Posture.

[6] Steingrebe H., Spancken S., Sell S., Stein T. (2023). Effects of hip osteoarthritis on lower body joint kinematics during locomotion tasks: A systematic review and meta-analysis. Front. Bioeng. Biotechnol..

[7] Diamond L.E., Allison K., Dobson F., Hall M. (2018). Hip joint moments during walking in people with hip osteoarthritis: A systematic review and meta-analysis. Osteoarthr. Cartil..

[8] Courtine G., Schieppati M. (2003). Human walking along a curved path. I. Body trajectory, segment orientation and the effect of vision. Eur. J. Neurosci..

[9] Hicheur H., Vieilledent S., Berthoz A. (2005). Head motion in humans alternating between straight and curved walking path: Combination of stabilizing and anticipatory orienting mechanisms. Neurosci. Lett..

[10] Dixon P.C., Stebbins J., Theologis T., Zavatsky A.B. (2013). Spatio-temporal parameters and lower-limb kinematics of turning gait in typically developing children. Gait Posture.

[11] Krafft F.C., Eckelt M., Köllner A., Wehrstein M., Stein T., Potthast W. (2015). Reproducibility of spatio-temporal and dynamic parameters in various, daily occurring, turning conditions. Gait Posture.

[12] Glaister B.C., Bernatz G.C., Klute G.K., Orendurff M.S. (2007). Video task analysis of turning during activities of daily living. Gait Posture.

[13] Leach J.M., Mellone S., Palumbo P., Bandinelli S., Chiari L. (2018). Natural turn measures predict recurrent falls in community-dwelling older adults: A longitudinal cohort study. Sci. Rep..

[14] Hase K., Stein R.B. (1999). Turning strategies during human walking. J. Neurophysiol..

[15] Taylor M.J.D., Dabnichki P., Strike S.C. (2005). A three-dimensional biomechanical comparison between turning strategies during the stance phase of walking. Hum. Mov. Sci..

[16] Katz J.N., Arant K.R., Loeser R.F. (2021). Diagnosis and Treatment of Hip and Knee Osteoarthritis: A Review. JAMA.

[17] Steultjens M.P., Dekker J., van Baar M.E., Oostendorp R.A., Bijlsma J.W. (2000). Range of joint motion and disability in patients with osteoarthritis of the knee or hip. Rheumatology.

[18] Tateuchi H., Tsukagoshi R., Fukumoto Y., Akiyama H., So K., Kuroda Y., Ichihashi N. (2014). Compensatory turning strategies while walking in patients with hip osteoarthritis. Gait Posture.

[19] Sedgman R., Goldie P., Iansek R. (1994). Development of a measure of turning during walking. Advancing Rehabilitation, Inaugural Conference of the Faculty of Health Sciences, Melbourne, Australia, 2–4 November 1994.

[20] Shi Y., Xu J., Zhang H., Jia L., Qin Y. (2022). Empirical investigation on turning behavior of passengers in subway station. Phys. A Stat. Mech. Its Appl..

[21] Dixon P.C., Jacobs J.V., Dennerlein J.T., Schiffman J.M. (2018). Late-cueing of gait tasks on an uneven brick surface impacts coordination and center of mass control in older adults. Gait Posture.

[22] Nolasco L.A., Silverman A.K., Gates D.H. (2019). Whole-body and segment angular momentum during 90-degree turns. Gait Posture.

[23] Deathe A.B., Miller W.C. (2005). The L Test of Functional Mobility: Measurement Properties of a Modified Version of the Timed “Up & Go” Test Designed for People with Lower-Limb Amputations. Phys. Ther..

[24] Xu D., Carlton L.G., Rosengren K.S. (2004). Anticipatory postural adjustments for altering direction during walking. J. Mot. Behav..

[25] Härtel T., Hermsdorf H. (2006). Biomechanical modelling and simulation of human body by means of DYNAMICUS. J. Biomech..

[26] Harrington M.E., Zavatsky A.B., Lawson S.E.M., Yuan Z., Theologis T.N. (2007). Prediction of the hip joint centre in adults, children, and patients with cerebral palsy based on magnetic resonance imaging. J. Biomech..

[27] Nérot A., Nicholls M. (2017). Clinical study on the unloading effect of hip bracing on gait in patients with hip osteoarthritis. Prosthet. Orthot. Int..

[28] Faul F., Erdfelder E., Lang A.-G., Buchner A. (2007). G*Power 3: A flexible statistical power analysis program for the social, behavioral, and biomedical sciences. Behav. Res. Methods.

[29] R Core Team R: A Language and Environment for Statistical Computing. https://www.R-project.org/.

[30] Schache A.G., Blanch P.D., Dorn T.W., Brown N.A.T., Rosemond D., Pandy M.G. (2011). Effect of running speed on lower limb joint kinetics. Med. Sci. Sports Exerc..

[31] Rutherford D.J., Moreside J., Wong I. (2015). Hip joint motion and gluteal muscle activation differences between healthy controls and those with varying degrees of hip osteoarthritis during walking. J. Electromyogr. Kinesiol..

[32] Zügner R., Tranberg R., Lisovskaja V., Kärrholm J. (2018). Different reliability of instrumented gait analysis between patients with unilateral hip osteoarthritis, unilateral hip prosthesis and healthy controls. BMC Musculoskelet. Disord..

[33] Hurwitz D.E., Hulet C.H., Andriacchi T.P., Rosenberg A.G., Galante J.O. (1997). Gait compensations in patients with osteoarthritis of the hip and their relationship to pain and passive hip motion. J. Orthop. Res..

[34] Yamaguchi T., Okamoto R., Hokkirigawa K., Masani K. (2018). Decrease in required coefficient of friction due to smaller lean angle during turning in older adults. J. Biomech..

[35] Roelker S.A., Caruthers E.J., Baker R.K., Pelz N.C., Chaudhari A.M.W., Siston R.A. (2017). Interpreting Musculoskeletal Models and Dynamic Simulations: Causes and Effects of Differences Between Models. Ann. Biomed. Eng..

[36] Falisse A., van Rossom S., Gijsbers J., Steenbrink F., van Basten B.J.H., Jonkers I., van den Bogert A.J., de Groote F. (2018). OpenSim Versus Human Body Model: A Comparison Study for the Lower Limbs During Gait. J. Appl. Biomech..

[37] Akram S.B., Frank J.S., Chenouri S. (2010). Turning behavior in healthy older adults: Is there a preference for step versus spin turns?. Gait Posture.

[38] Bergsma B., Hulleman D.N., Wiedemeijer M.M., Otten E. (2021). Foot placement variables of pedestrians in community setting during curve walking. Gait Posture.

[39] Xu D., Chow J.W., Wang Y.T. (2006). Effects of turn angle and pivot foot on lower extremity kinetics during walk and turn actions. J. Appl. Biomech..

[40] Foucher K.C. (2017). Sex-specific hip osteoarthritis-associated gait abnormalities: Alterations in dynamic hip abductor function differ in men and women. Clin. Biomech..

