# Supplementary material for: Mobility Requirements and Joint Loading during Straight Walking and 90° Turns in Healthy Older People and Those with Hip Osteoarthritis
Source: J Clin Med. 2024 Aug 25;13(17):5021. doi: 10.3390/jcm13175021 (PMC11396374; doi:10.3390/jcm13175021)
Supplement: Supplementary file 1 [file jcm-13-05021-s001.zip › jcm-3020908-supplementary.pdf]

Table S1. P-values of Shapiro-Wilk tests, Levene tests and mixed-model ANOVA. HOA = hip osteoarthritis group; IC = initial contact; TO = toe off; COM = centre of mass; ROM = range of motion.

	Shapiro-Wilks Test (p)						Levene-Test (p)	Mixed-Model ANOVA (p)		
	Healthy			HOA			Group	Main effects		Interaction effect
Parameter	Straight	Step Turn	Spin Turn	Straight	Step Turn	Spin Turn		Group	Movement	Group*Movement task
COM velocity IC [m/s]	0.125	0.379	0.995	0.906	0.333	0.241	0.497	0.187	<0.001	0.333
COM velocity TO [m/s]	0.029	0.363	0.654	0.775	0.139	0.322	0.837	0.123	<0.001	0.139
Stance phase duration [s]	0.710	0.036	0.666	0.348	0.271	0.003	0.198	0.292	<0.001	0.271
Peak hip flexion [°]	0.768	0.345	0.910	0.269	0.206	0.942	0.564	0.464	<0.001	0.206
Peak hip extension [°]	0.562	0.063	0.532	0.069	0.141	0.897	0.311	0.002	<0.001	0.141
Sagittal hip ROM [°]	0.242	0.794	0.613	0.332	0.041	0.364	0.128	0.001	<0.001	0.041
Peak hip abduction [°]	0.531	0.147	0.886	0.258	0.001	0.282	0.425	0.997	<0.001	0.001
Peak hip adduction [°]	0.353	0.051	0.513	0.320	<0.001	0.988	0.206	0.215	<0.001	<0.001
Frontal hip ROM [°]	0.369	0.100	0.163	0.059	0.081	0.088	0.317	0.154	<0.001	0.081
Peak hip ext. rotation [°]	0.592	0.479	0.969	0.097	0.281	0.135	0.089	0.849	<0.001	0.281
Peak hip int. rotation [°]	0.896	0.939	0.948	0.042	0.046	0.314	0.244	0.099	<0.001	0.046
Transverse hip ROM [°]	0.632	0.560	0.122	0.703	0.006	0.256	0.379	<0.001	<0.001	0.006
Peak hip extension moment [Nm/kg]	<0.001	0.002	0.001	0.181	0.851	0.001	0.852	0.349	0.006	0.851
Peak hip flexion moment [Nm/kg]	<0.001	0.395	0.537	0.653	0.493	0.423	0.132	0.823	<0.001	0.493
Peak hip abduction moment [Nm/kg]	0.149	0.003	0.002	0.048	0.740	0.001	0.616	0.637	<0.001	0.740
Peak hip adduction moment [Nm/kg]	0.002	0.001	0.001	0.088	0.730	0.002	0.595	0.667	<0.001	0.730
Peak hip ext. rotation moment [Nm/kg]	0.859	0.012	0.017	0.092	0.175	0.039	0.634	0.554	<0.001	0.175
Peak hip int. rotation moment [Nm/kg]	<0.001	0.839	<0.001	0.244	0.198	0.003	0.322	0.319	<0.001	0.198

Table S2. Bonferroni corrected p-values from pairwise comparisons of significant ANOVA main effects for movement task using dependent sample t-tests.

	Bonferroni corrected dependent sample t-tests (p)		
**Parameter**	**Straight vs. Step Turn**	**Straight vs. Spin Turn**	**Step Turn vs. Spin Turn**
COM velocity IC [m/s]	<0.001	<0.001	0.364
COM velocity TO [m/s]	<0.001	<0.001	1.000
Stance phase duration [s]	<0.001	<0.001	<0.001
Peak hip flexion [°]	<0.001	0.001	<0.001
Peak hip extension [°]	<0.001	<0.001	1.000
Sagittal hip ROM [°]	<0.001	<0.001	<0.001
Peak hip abduction [°]	<0.001	<0.001	<0.001
Peak hip adduction [°]	<0.001	<0.001	<0.001
Frontal hip ROM [°]	<0.001	1.000	<0.001
Peak hip ext. rotation [°]	<0.001	<0.001	<0.001
Peak hip int. rotation [°]	<0.001	<0.001	0.162
Transverse hip ROM [°]	<0.001	<0.001	<0.001
Peak hip extension moment [Nm/kg]	<0.001	<0.001	1.000
Peak hip flexion moment [Nm/kg]	<0.001	<0.001	1.000
Peak hip abduction moment [Nm/kg]	<0.001	<0.001	<0.001
Peak hip adduction moment [Nm/kg]	0.910	<0.001	<0.001
Peak hip ext. rotation moment [Nm/kg]	0.004	<0.001	<0.001
Peak hip int. rotation moment [Nm/kg]	<0.001	<0.001	<0.001
